# The complete mitochondrial genome of *Acrossocheilus yunnanensis* (Teleostei: Cypriniformes: Cyprinidae) and its phylogenetic position

**DOI:** 10.1080/23802359.2019.1688100

**Published:** 2019-11-14

**Authors:** Chuntao Li, Kaiqin Chen, Zhengmin Qian, Boping Zeng, Peiyong Song, Zili Jiang

**Affiliations:** College of Biology and Agriculture, Zunyi Normal College, Zunyi, Guizhou, China

**Keywords:** Mitochondrial DNA, *Acrossocheilus yunnanensis*, Cyprinidae, phylogeny

## Abstract

The complete mitochondrial genome of *Acrossocheilus yunnanensis* was determined in this study. It contained 13 protein-coding genes (PCGs), 22 tRNA, 2 rRNAs, and a control region with the base composition 31.47% A, 27.83% C, 24.65% T, and 16.05% G. Here we compared this newly determined mitogenome with another one from the same species reported before. The variable sites and the genetic distances between the two mitogenomes were 134 bp and 0.8%. Sixty-five variable sites occurred in the PCGs. The results from the phylogenetic analysis showed that the genus *Acrossocheilus* is not a monophyletic group and *Acrossocheilus yunnanensis* demonstrates a close relationship with *Acrossocheilus monticola*.

*Acrossocheilus yunnanensis* (Cypriniformes: Cyprinidae: Barbinae) is mainly distributed in the Pearl River and Yangtze River drainages in East Asia (Froese and Pauly 2019). It is an important food source in this region. Here the complete mitochondrial genome sequence of *A. yunnanensis* was determined (GenBank accession No. MN395748) and was compared with another *A. yunnanensis* mitogenome data reported before (Wu et al. 2016). The specimens (Voucher No. 20190528005) were collected from Chishui City (28.35 N, 105.42 E), Guizhou Province, China, and were stored in the museum of the Freshwater Fish Research Laboratory (Zunyi Normal College, Guizhou, China). Primers used in this study and methods for collecting DNA sequences followed the procedures outlined in Wang et al. (2011).

The complete mitochondrial genome of *A. yunnanensis* was a circular molecule with 16,590 bp in length. It contained 13 PCGs, 22 tRNA genes, 2 rRNA genes, and a control region. Most of the genes were encoded on H-strand, while *ND6* and 8 tRNA genes were encoded on L-strand. The overall nucleotide composition was 31.11% A, 24.81% T, 27.80% C, and 16.27% G, with a slight AT bias. All the mitochondrial PCGs in the *A. yunnanensis* use the standard ATG start codon, except for *ND3*, which utilizes GTG. One PCGs contain TAA stop codon, four contain TAG stop codon and four contain TGA stop codon, while four contain the incomplete stop codon T–.

Comparing with another mitogenome of *A. yunnanensis*, the length of it was 16,596 bp (accession No. KR062067; Wu et al. 2016). The genetic distance between the two mitogenomes was 0.8%. 11 indels and 134 variable sites exist in between the two mitogenomes. Among these variable sites, 77 transitions (57%) and 57 transversions (43%) were found. There were 65 variable sites appeared in the PCGs and 7 sites in control region. The other 62 variable sites occurred in *12S rRNA*, *16S rRNA*, and tRNAs.

To confirm the phylogenetic position of *A. yunnanensis* among the genus *Acrossocheilus*, phylogenetic analysis based on the complete mitogenome using maximum-likelihood (ML) and Bayesian methods (BI) were conducted using IQ-TREE (Nguyen et al. 2015; Hoang et al. 2018) and MrBayes 3.2.6 (Ronquist et al. 2012), respectively. The ModelTest was used to calculate the optimal nucleotide substitution model (Posada and Crandall 1998). Phylogenetic relationships inferred by the BI and ML methods were congruent, the ML tree is given in Figure. 1. The results from the analyses show that the genus *Acrossocheilus* is not a monophyletic group and *A. yunnanensis* demonstrates a close relationship with *Acrossocheilus monticola*.

**Figure 1.**
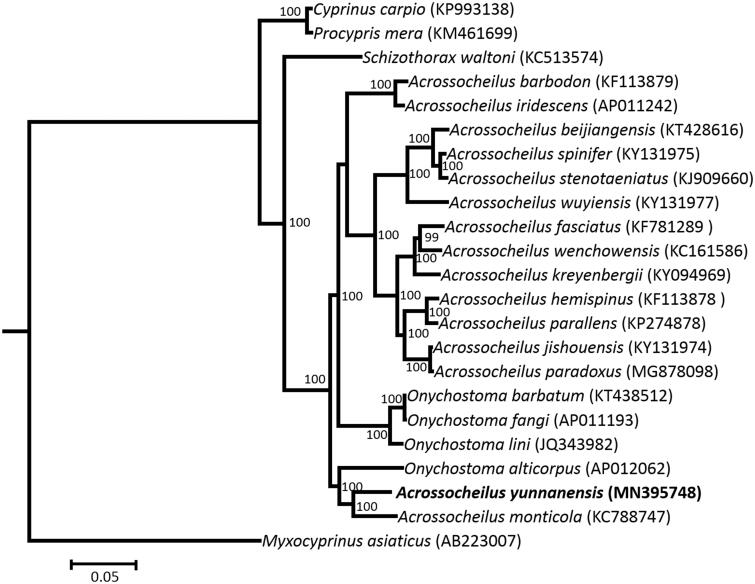
Phylogenetic tree of *Acrossocheilus yunnanensis* and the other 21 cyprinid species based on maximum-likelihood (ML) method. The ML bootstrap values are shown at the nodes.

## References

[CIT0001] Froese R, Pauly D, editors. 2019 FishBase. World Wide Web electronic publication; [accessed 2019 September 2]. http://www.fishbase.org, version (08/2019).

[CIT0002] Hoang DT, Chernomor O, von Haeseler A, Minh BQ, Vinh LS 2018 UFBoot2: improving the ultrafast bootstrap approximation. Mol Biol Evol. 35(2):518–522. 29077904 10.1093/molbev/msx281 PMC5850222

[CIT0003] Nguyen LT, Schmidt HA, von Haeseler A, Minh BQ 2015 IQ-TREE: a fast and effective stochastic algorithm for estimating maximum-likelihood phylogenies. Mol Biol Evol. 32(1):268–274. 25371430 10.1093/molbev/msu300 PMC4271533

[CIT0004] Posada D, Crandall KA 1998 MODELTEST: testing the model of DNA substitution. Bioinformatics. 14(9):817–818. 9918953 10.1093/bioinformatics/14.9.817

[CIT0005] Ronquist F, Teslenko M, van der Mark P, Ayres DL, Darling A, Höhna S, Larget B, Liu L, Suchard MA, Huelsenbeck JP, et al. 2012 MrBayes 3.2: efficient Bayesian phylogenetic inference and model choice across a large model space. Syst Biol. 61(3):539–542. 22357727 10.1093/sysbio/sys029 PMC3329765

[CIT0006] Wang J, Li P, Zhang Y, Peng Z 2011 The complete mitochondrial genome of Chinese rare minnow, *Gobiocypris rarus* (Teleostei: Cypriniformes). Mitochondrial DNA. 22(5–6):178–180. 22165834 10.3109/19401736.2011.636441

[CIT0007] Wu JY, Huang SQ, Du ZJ, Xie M, Zhu G, Wang Q, Jiang Y, He T, Ren H, Xhang Y, et al. 2016 Complete mitochondrial genome of *Acrossocheilus yunnanensis* (Cypriniformes, Barbinae, Acrossocheilus). Mitochondrial DNA Part A. 27(4):2623–2624. 10.3109/19401736.2015.1041124 26024134

